# Modeling amyloid therapy potential on a population level: An adaptation of the MISCAN‐Dementia model

**DOI:** 10.1002/alz70860_105453

**Published:** 2025-12-23

**Authors:** Chiara C. Brück, Frank J. Wolters, Everard G.B. Vijverberg, Inge M.C.M. de Kok

**Affiliations:** ^1^ Department of Public Health, Erasmus MC University Medical Center, Rotterdam, Netherlands; ^2^ Department of Epidemiology, Erasmus University Medical Center, Rotterdam, Netherlands; ^3^ Alzheimer Center, Department of Neurology, Amsterdam UMC, Vrije Universiteit Amsterdam, Amsterdam Neuroscience, Amsterdam, Netherlands

## Abstract

**Background:**

Amyloid‐targeting therapies for Alzheimer's disease are currently under consideration or approved for use in various countries. However, the population‐wide impact of these therapies and eligible population remain unclear. This project aims to adapt the microsimulation model MISCAN‐Dementia to estimate the eligible population and population‐wide impact of amyloid‐targeting therapies.

**Method:**

We utilize the MISCAN‐Dementia microsimulation model, which we previously developed to predict mild cognitive impairment (MCI) and dementia outcomes (e.g. incidence, prevalence, utility, costs) in the general population. For the purpose of this study, we adapt the model to include age‐ and dementia‐stage specific parameters for amyloid positivity, tailored to the target population with MCI or mild Alzheimer's dementia. Furthermore, we validate the correctness of the model's MCI stage with external data and add co‐morbidities (e.g. microbleeds, medication use, APOE e4/e4) that prohibit the use of amyloid‐targeting therapies. Finally, the eligible population and population‐wide impact of amyloid‐targeting therapies is estimated.

**Result:**

Validating the MCI stage presents multiple methodological difficulties. First, while there is ongoing research aimed at better understanding MCI and its progression to dementia, there is relatively limited evidence on its age‐specific incidence and prevalence. Second, an MCI diagnosis relies on performance on memory tests relative to age‐appropriate norms, leading to different cutoffs across populations. Third, stable MCI or reversion back to normal cognition is fairly common, possibly resulting in discrepancies between incidence and prevalence within the same population. Consequently, data on MCI incidence and prevalence in literature is very heterogenous. Due to its high‐quality population‐based data and long‐term follow‐up, we use published data of the Mayo Clinic Study of Aging for the validation. Finally, other adaptations (amyloid status and co‐morbidities) of MISCAN‐Dementia are ongoing.

**Conclusion:**

We are adapting the MISCAN‐Dementia model to estimate the population‐wide impact of amyloid‐targeting therapies. In this presentation, we will present the model changes and methodological considerations we have encountered, as well as first estimates of the eligible population. To capture the impact of amyloid‐targeting therapies fully, the next steps include estimating the population‐wide harms and benefits of amyloid‐targeting therapies.

